# Intraductal Papillary Neoplasm of the Bile Duct Posing Diagnostic and Therapeutic Challenges

**DOI:** 10.7759/cureus.73501

**Published:** 2024-11-12

**Authors:** Karthik Chalamalasetti, Prabhakaran R, Senthil Kumar P, Sugumar Chidambaranathan

**Affiliations:** 1 Surgical Gastroenterology, Kalaignar Centenary Super Specialty Hospital, Chennai, IND; 2 Surgical Gastroenterology, Madras Medical College, Chennai, IND

**Keywords:** bile duct tumour, biliary papillomatosis, intraductal papillary neoplasm of the bile duct, mucinous cystic neoplasm, s: cholangiocarcinoma

## Abstract

Intraductal papillary neoplasms of the bile duct are rare tumors with fibrovascular stalks arising from the bile duct. It is often difficult to diagnose preoperatively, especially from mucinous cystic neoplasms. The incidence is more common in Asian countries and rare in Western countries. We report a series of patients with Intraductal papillary neoplasms of the bile duct and describe challenges faced during diagnosis and subsequent management. All the patients differed in clinical and radiological presentation and hence required different approaches to management. The first two cases presented as a space-occupying lesion of the liver, and the third one was a periampullary lesion. All were managed with surgical resection. These neoplasms are uncommon in India and often pose diagnostic and therapeutic difficulties. As the spectrum of presentation varies from benign neoplasm to invasive carcinoma, surgical resection should be performed as it offers the possibility of a cure.

## Introduction

Intraductal papillary neoplasm of the bile duct (IPNB) is defined by the WHO as a “papillary or villous neoplasm covering delicate fibrovascular stalks occurring in the bile ducts” [1]. IPNB was added to the 2010 World Health Organization (WHO) classification, which was classified separately from mucinous cystic neoplasm (MCN, including biliary cystadenoma/cystadenocarcinoma); MCN differs from IPNB where there is no communication with the biliary tree in MCN and has ovarian stroma [1]. These tumors are mostly benign but may exhibit a variable degree of atypia and progress to carcinoma. Owing to the wide range of clinical presentations, this tumor is often difficult to identify preoperatively. Here are case reports described that posed diagnostic dilemmas and therapeutic challenges.

## Case presentation

Case 1

A 42-year-old female presented with features of obstructive jaundice. There was no history of abdominal pain, fever, loss of appetite, or weight loss. On examination, there was icterus and hepatomegaly with no other signs of chronic liver disease. A liver function test (Table 1) demonstrated total bilirubin of 14.5 mg/dl (normal range 0.3-1.3 mg/dl) and direct bilirubin of 11.5 mg/dl, (normal range 0.1-0.4mg/dl), Aspartate transaminase (AST) of 103 IU/L (normal range 12-38 IU/L), alanine transaminase (ALT) of 58 IU/L (normal range 4-41 IU/L), and alkaline phosphatase (ALP) of 566 IU/L (normal range 35-130 IU/L). She tested positive for Hepatitis B infection with HBV DNA levels of 602 copies/ml. 

**Table 1:** Liver function tests AST: Aspartate transaminase; ALT: Alanine transaminase; ALP: Alkaline phosphatase; HBV: Hepatitis B Virus

Liver function test	Patient results	Normal range
Total bilirubin (mg/dl)	14.5	0.3-1.3
Direct bilirubin (mg/dl)	11.5	0.1-0.4
AST (IU/liter)	103	12-38
ALT (IU/liter)	58	4-41
ALP (IU/liter)	566	35-130
HBV DNA level (copies/ml)	602	<10,000

**Figure 1:**
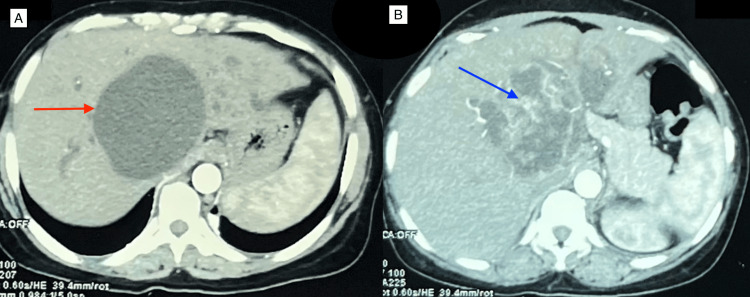
CECT abdomen (A) Image showing a large cystic lesion (red arrow) in segments 2, 3 and 4 extending to segments 5 and 8 of the liver. (B) The solid components show a significant contrast enhancement (blue arrow). CECT: Contrast-enhancement computed tomography

Upper gastrointestinal endoscopy demonstrated an extraneous impression along the lesser curvature with a normal ampulla. Contrast-enhanced computed tomography (CECT) abdomen (Figure 1) revealed a well-defined cystic lesion with few solid components of size 8.7 x 7 x 7.4 cm in segments 2,3 and 4 extending to segments 5 & 8 of the liver, compressing the common hepatic duct (CHD), causing intrahepatic biliary radical dilation (IHBRD). The solid components showed a significant enhancement, and the common bile duct (CBD) was dilated (9 mm) with a heterodense lesion at the opening of CBD into the duodenum of size 8 x 8 mm. 

**Figure 2:**
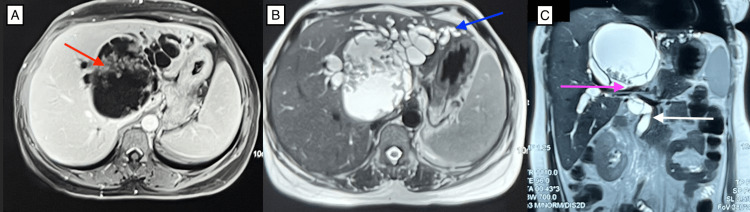
MRI abdomen (A) MRI abdomen showing T1 hypointense cystic lesion with papillary excrescences (red arrow). (B) The lesion is hyperintense in the T2 phase with dilatation of the left biliary system (blue arrow). (C) The cyst is compressing the confluence of the right and left hepatic ducts (pink arrow). The dilated common bile duct can also be appreciated (white arrow).

MRI of the abdomen (Figure 2) revealed similar findings of a well-defined T1 hypointense and T2 hyperintense (9.6 (anteroposterior) x 8 (transverse) x 7.8 (cranio-caudal) cms) solitary cystic lesion with multiple internal septations and papillary excrescences noted compressing the confluence of the right hepatic duct and the left hepatic duct with communicating with bile ducts. There was contrast enhancement of the peripheral solid papillary excrescence. However, the lesion at the distal common bile duct couldn’t be appreciated. 

**Figure 3:**
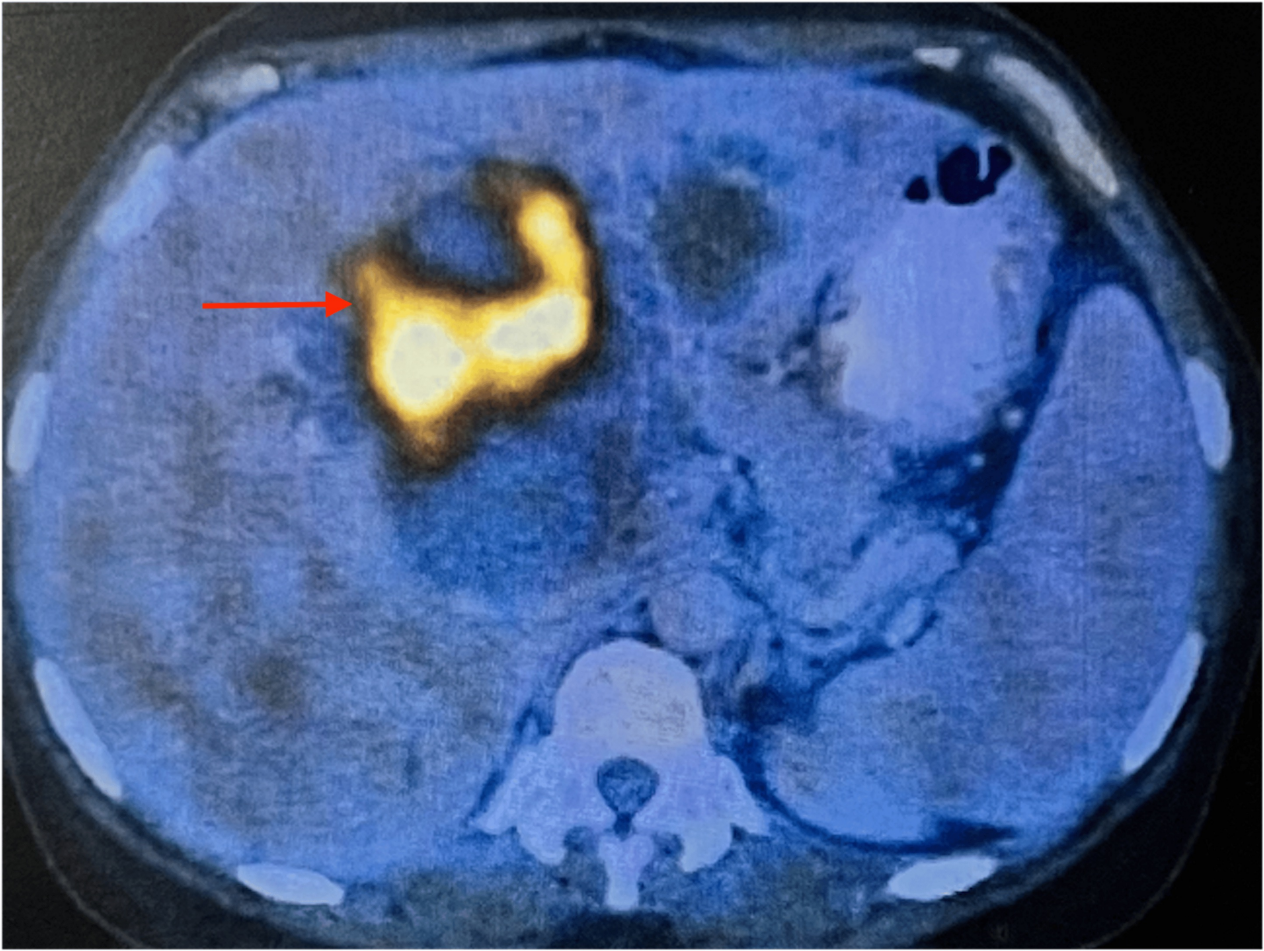
PET/CT scan The image shows a well-defined cystic lesion in the liver with metabolically active papillary excrescences (red arrow). PET/CT scan: positron emission tomography-computed tomography scan

She also underwent a positron emission tomography-computed tomography scan (PET/CT) to rule out a second primary tumor, as CECT showed a second lesion at the ampulla. PET/CT showed the lesion had multiple irregular metabolically active enhancing solid mural papillary projections (maximum standardized uptake value (SUVmax) = 11.9) with no lesion or uptake in the distal CBD (Figure 3).

**Figure 4:**
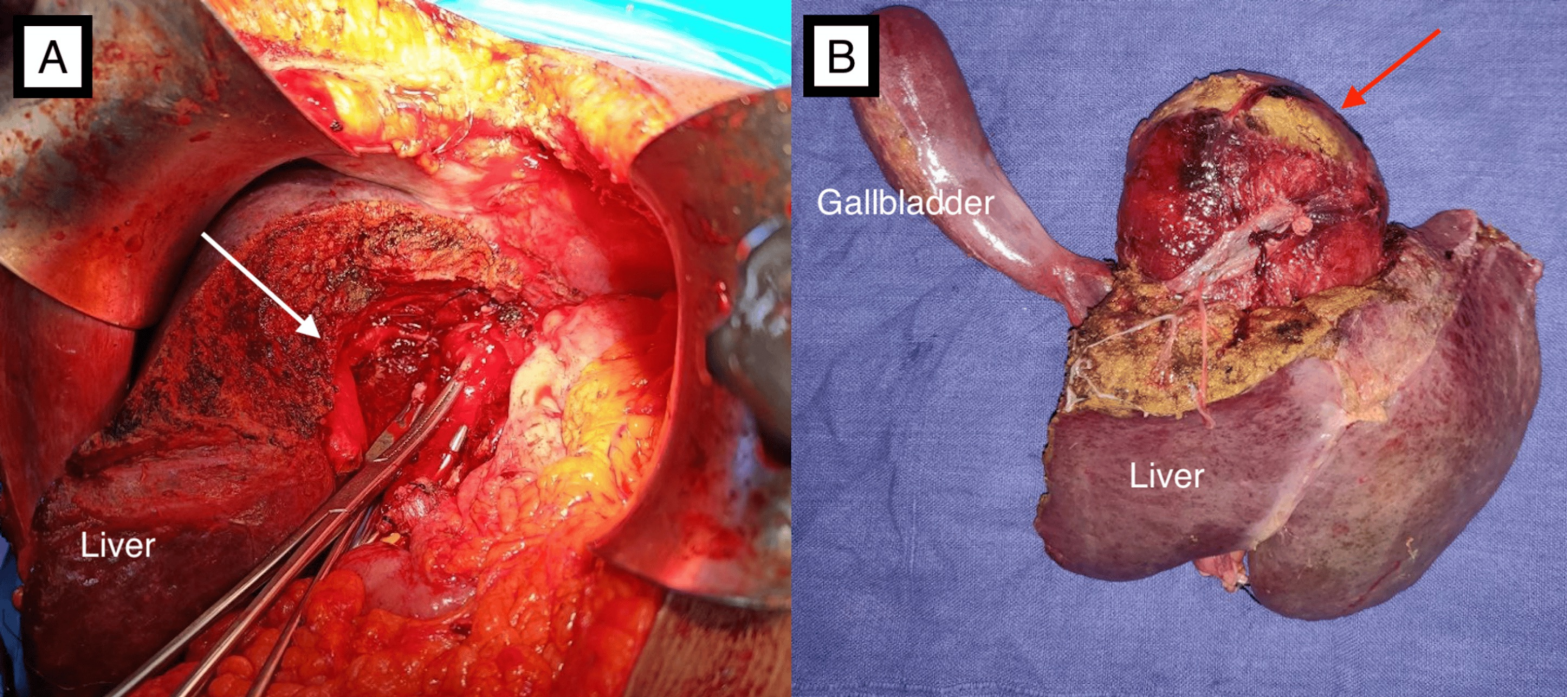
Operative images (A) Image after left hepatectomy along with cyst excision, the right anterior portal pedicle can be appreciated (white arrow). (B) Postoperative specimen of the cyst (red arrow) along with resected liver parenchyma.

**Figure 5:**
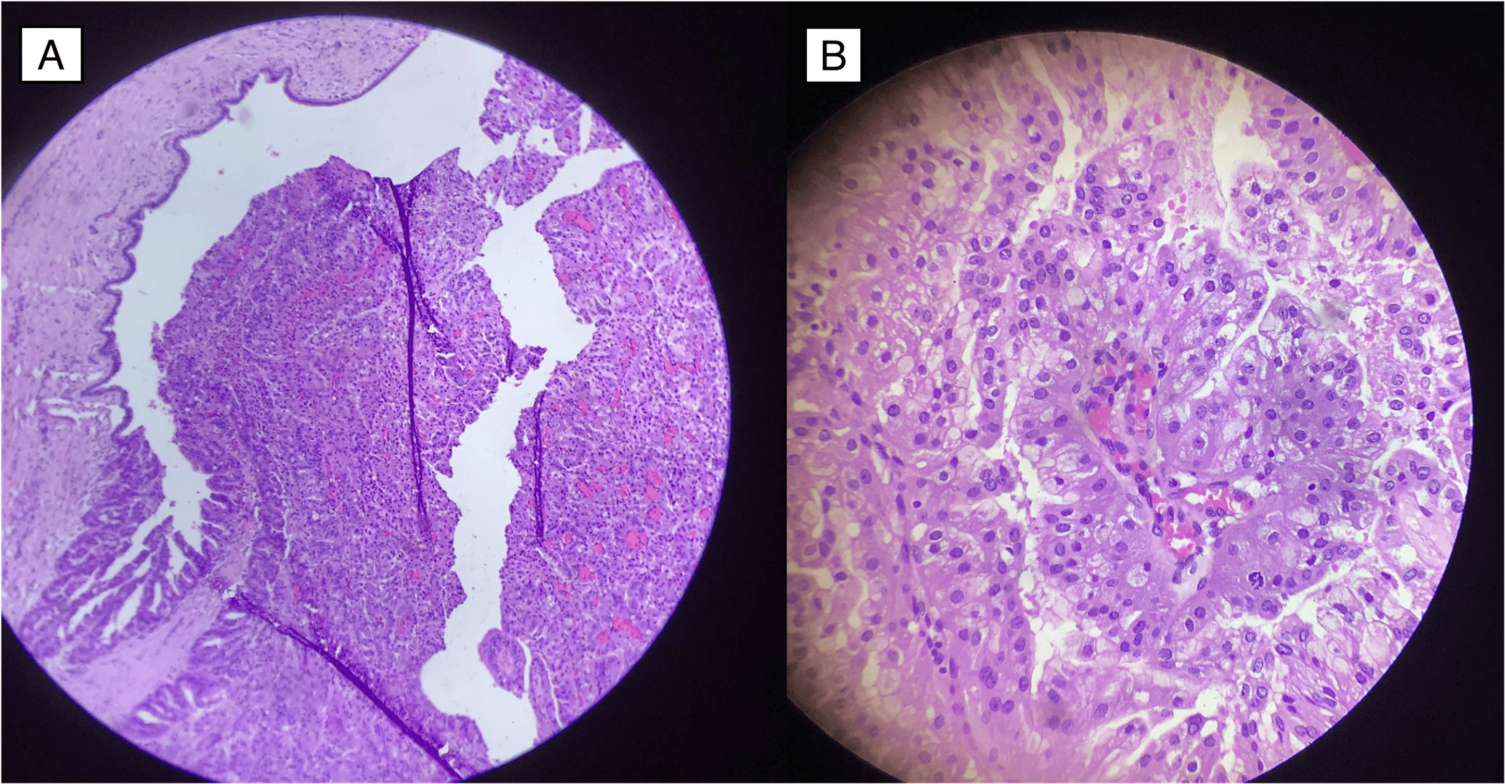
Postoperative histopathology (A) Tumor arising from the bile duct (B) High power field showing papillary architecture

She subsequently underwent endoscopic retrograde cholangiopancreatography (ERCP) with CBD stenting for rising bilirubin, and the decision was taken to opt for surgical resection. Intraoperatively, there was a cystic lesion of size around 10 x 9 cm at the hilum of the liver, but fortunately, it engulfed only the left hepatic duct, left hepatic artery, and the left portal vein. The lesion involved segments 2,3 and 4, extending onto segments 5,8 of the liver (Figure 4). Mucinous material was noted in the distal CBD, causing proximal dilatation of the bile duct. As the liver was nodular and fibrotic, to preserve the liver parenchyma, a left hepatectomy along with total excision of the cyst was done. The extrahepatic bile duct was excised, and the right cholangiojejunosotomy was done. Her postoperative histopathology revealed intraductal papillary neoplasm with low-grade dysplasia (Figure 5). She is on regular follow-up, and one year after surgery, the patient is symptom-free with no evidence of recurrence.

Case 2 

A 55-year-old female presented with pain in the right upper abdomen for two months. There was no history of yellowish discoloration of the eyes, fever, loss of appetite, or loss of weight. Her comorbidities included chronic kidney disease, for which she was on medication. Abdominal examination was unremarkable. Liver function tests were within normal limits, and viral markers were negative. Cancer antigen 19-9 (CA 19-9) was 396 U/ml, and an upper gastrointestinal endoscopy was normal.

**Figure 6:**
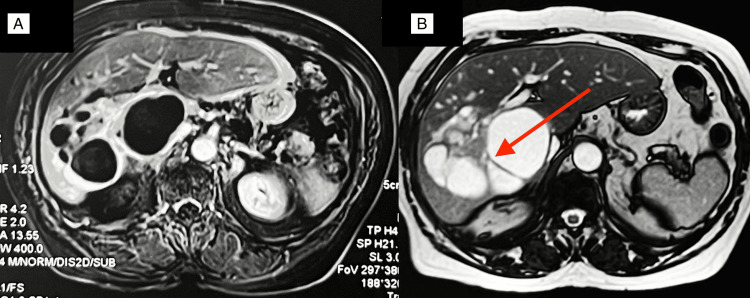
MRI abdomen (A) MRI angiogram in the T1 phase shows a peripherally enhancing hypointense cystic lesion involving segments 6, 7 and 8 of the liver. (B) The lesion is hyperintense in the T2 phase with multiple internal septations (red arrow).

CECT abdomen showed a multilocular cystic lesion of size 6.9 x 9 x 7.2 cm involving segments 6, 7, and 8 of the liver, causing upstream dilatation of right biliary radicles, suggesting the possibility of biliary cystadenoma. MRI abdominal angiogram revealed similar findings with T1 hypointense and T2 hyperintense large cystic lesion (with low apparent diffusion coefficient (ADC)) with solid components and internal septation. The lesion was enhanced peripherally with the contrast, and upstream right intrahepatic biliary radicle dilatation was evident (Figure 6). In view of the above findings, a probable diagnosis of biliary cystadenocarcinoma was made.

**Figure 7:**
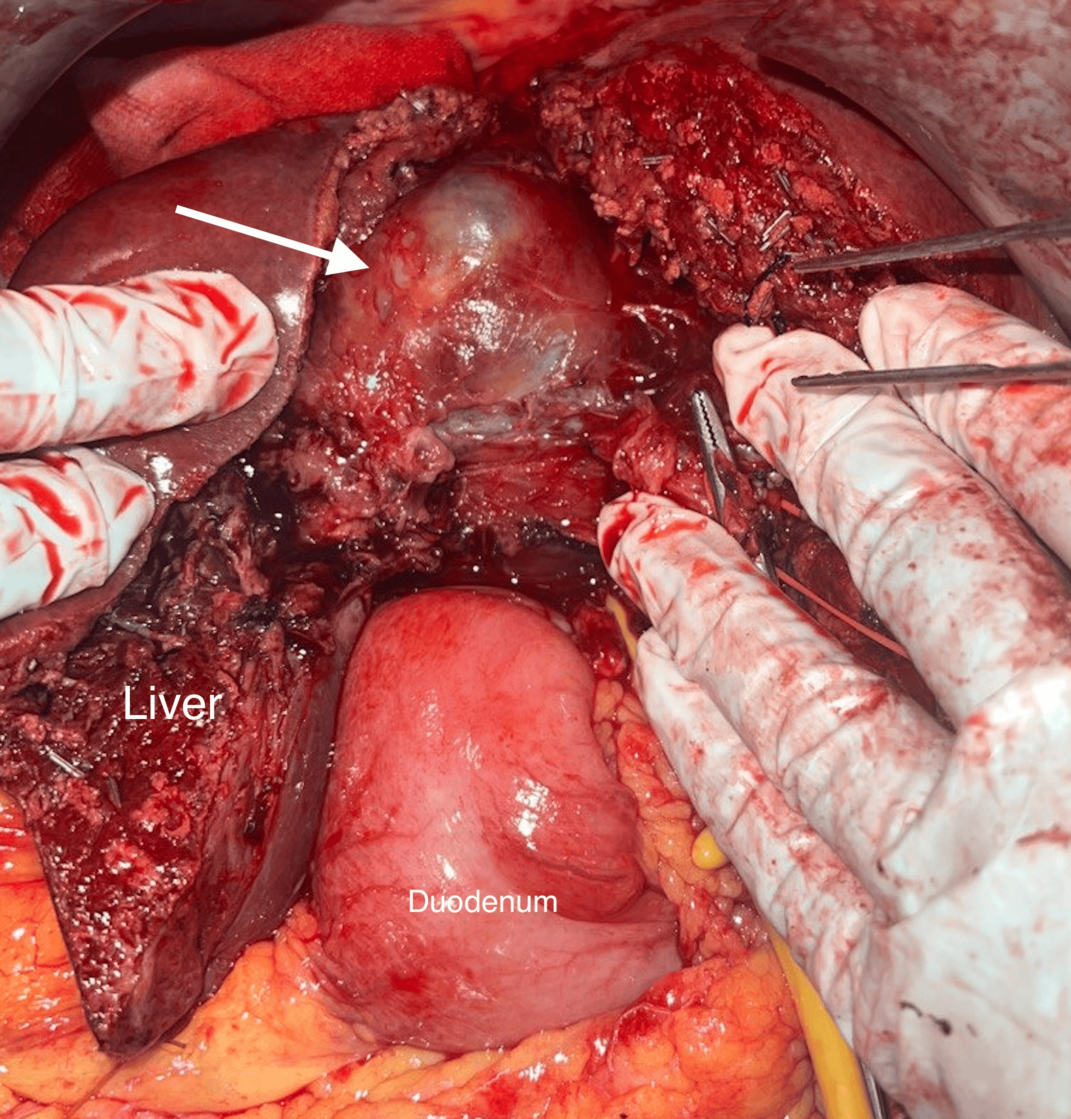
Intraoperative image Right hepatectomy along with cyst (white arrow) excision.

**Figure 8:**
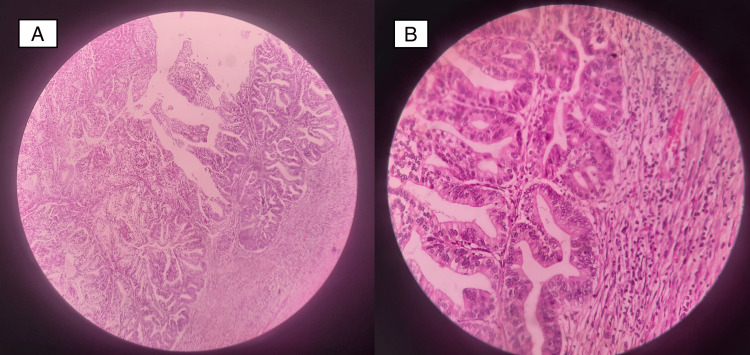
Postoperative histopathology (A) Histopathology showing papillary architecture arising from the bile duct. (B) High power field of the tumor

An initial staging laparoscopy ruled out evidence of metastasis, and intraoperatively, a mass of size 10 x 8 cm involving the segments 6, 7, and 8 with an abutment to the inferior vena cava was noted. A decision was taken to proceed with the right hepatectomy, and during parenchymal transection, the lesion was found to have an extension into segment 4 (Figure 7). Right hepatectomy along with cyst excision was done. Postoperative histopathology was consistent with intraductal papillary neoplasm of the bile duct with invasive carcinoma (pT1bN0M0); the resected margin showed features of tumor infiltration. A tumor thrombus was also noted in the bile duct. Immunohistochemistry showed strong membrane positivity for cytochrome 7 (CK7) (>90% tumor cells), cytochrome 20 (CK20)- focal membrane positivity in >20% tumor cells, cytochrome 19 (CK19)-focal positivity in 50-60% cells, and caudal type homeobox 2 (CDX2) was negative (Figure 8). Postoperative events were uneventful, and she was planning on adjuvant chemotherapy (Capecitabine) after the multidisciplinary team (MDT) discussion.

Case 3

A 78-year-old male presented with features of obstructive jaundice. A liver function test (Table 2) demonstrated total bilirubin of 19.6 mg/dl (normal range 0.3-1.3 mg/dl) and direct bilirubin of 13.2 mg/dl (normal range 0.1-0.4mg/dl), AST of 79 IU/L (normal range 12-38 IU/L), ALT of 40 IU/L (normal range 4-41 IU/L), and ALP of 349 IU/L (normal range 35-130 IU/L). Upper gastrointestinal endoscopy demonstrated a nodular lesion over the ampulla. The biopsy was negative for malignancy. 

**Table 2:** Liver function test AST: Aspartate transaminase; ALT: Alanine transaminase; ALP: Alkaline phosphatase

Liver function test	Patient results	Normal range
Total bilirubin (mg/dl)	19.6	0.3-1.3
Direct bilirubin (mg/dl)	13.2	0.1-0.4
AST (IU/liter)	79	12-38
ALT (IU/liter)	40	4-41
ALP (IU/liter)	349	35-130

**Figure 9:**
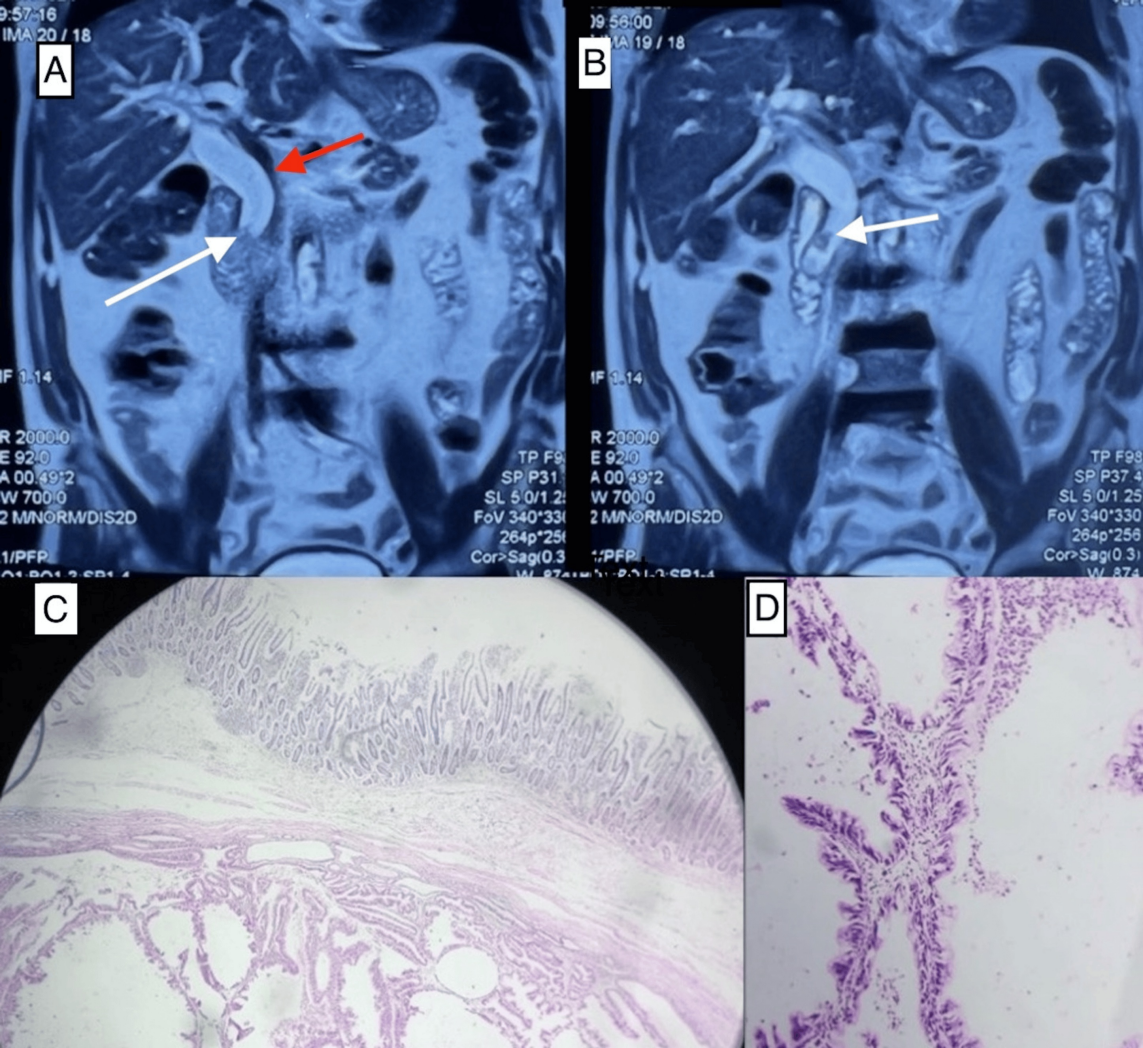
MRI imaging and postoperative histopathology (A) MRI imaging shows dilated CBD (red arrow) with a soft tissue lesion (white arrow). (B) Another polypoid soft tissue lesion in the distal bile duct can be appreciated (white arrow). (C) Postoperative histopathology shows overlying duodenal mucosa with underlying neoplasm from the bile duct. (D) High-power view of the tumor. CBD: Common bile duct

CECT and MRI of the abdomen showed two soft tissue lesions (15.6 x 12 mm and 13 x 12 mm, respectively) in distal CBD. The patient underwent pancreaticoduodenectomy, and postoperative histology showed features suggestive of Intraductal papillary neoplasm of the bile duct with invasive carcinoma (Figure 9). The patient underwent adjuvant chemotherapy after the MDT discussion.

## Discussion

Two major risk factors include hepatolithiasis and Clonorchis infection, hence its incidence is more in Asian countries such as Korea and Japan (9.9 to 30%) [1-3]. IPNB is uncommon, with an incidence of 7-11% of bile duct tumors in Western countries [1]. In most series, the median age of IPNB is 60-66 years, with a male predominance [4]. Common clinical presentations include pain (35%-88.5%), repeated episodes of acute cholangitis (5%-59%), and obstructive jaundice (20%-36%) [2]. Mucin or tumor emboli from the tumor often obstruct the biliary tract, which leads to jaundice in one-third of the patients [2,5], as was the case in the above-reported study. IPNB can further be classified into those producing mucin and those without. The non-mucin-secretin type was found to be more invasive compared to the mucin-secreting type. The mucin-secreting type has similarities to intraductal mucinous neoplasm of the pancreas, the IPNB-non-mucin-secreting type showed heterogeneous groups [6].

CT scan findings may vary from cystic lesions or gross dilatation of bile ducts. The most common findings include intraductal masses with intra and extrahepatic bile duct dilation (case study 1), and some may show masses with proximal ductal dilatation (case studies 2 and 3). MRI features include low or isointense in T1 and hyperintense in T2-weighted images. Lesions may show enhancement with contrast. It is often difficult to differentiate from biliary mucinous cystic neoplasms (MCNs) (cystadenoma/cystadenocarcinoma) preoperatively unless communication to the bile duct is evident [7].

IPNBs are classified into four types: pancreaticobiliary, intestinal, gastric, and oncolytic [8], based on hematoxylin and eosin (H&E) and immunohistochemistry of the mucin (MUC1, MUC2, MUC5AC, CK7, CK20). Pancreaticobiliary type has the worst prognosis [8]. IPNB may progress from low, intermediate, to high-grade, and it may also have an associated invasive carcinoma [4]. IPNB with high-grade intraepithelial neoplasia can progress to an intraductal growing cholangiocarcinoma [4].

Most of the tumors are benign, and surgical treatment offers the possibility of a cure [6,9]. Lymph node metastasis is rare in IPNB; if present, it confers a poor prognosis [2,8,9]. The recurrence rate at five years in benign IPNBs has been reported at nearly 20%, which rises to 60% in malignant cases as IPNB may be multifocal, and some lesions may often be missed with routine imaging. Invasive components occur in up to 74% [2,7]. Compared to cholangiocarcinoma, IPNB with an invasive component offers a better prognosis [9,10]. The five-year survival rate was reported to be up to 80% in non-invasive tumors [11]. Survival is related to the percentage and depth of the invasive component, lymphovascular invasion, and cellular atypia [10].

In the above first reported case, there was a diagnostic dilemma as obstructive jaundice was caused by the mucin produced from the IPNB, a lesion that itself is difficult to diagnose preoperatively. With a hepatitis B positive, a marginal liver status, and a tumor at the hilum of the liver, the decision was taken to remove the cyst with parenchymal preservation rather than a formal anatomical resection, as resection was the only chance for cure, apart from a liver transplant that is limited by scarce donor availability. However, the patient must be on follow-up for life as the reported recurrence is high and unpredictable for this rare neoplasm. The second case was IPNB with invasive carcinoma; technical difficulties in operative procedures precluded R0 resection, and the prognosis was poorer compared to the first case. However, she is planned for adjuvant chemotherapy and put on surveillance to decide upon the further plan of management. The third patient had a presentation like a periampullary carcinoma; this shows that the intraductal papillary neoplasm of the bile duct has a wide range of presentations, contributing to diagnostic and therapeutic difficulties.

## Conclusions

Intraductal papillary neoplasms of the bile duct are uncommon in India and often pose diagnostic and therapeutic difficulties. As the spectrum of presentation varies from benign neoplasm to invasive carcinoma, surgical resection should be performed as it offers the possibility of a cure.
